# ADAPT system is a dramatic advance in computer-assisted surgery for femoral trochanteric fractures

**DOI:** 10.1051/sicotj/2021056

**Published:** 2021-11-05

**Authors:** Tomotoshi Murakami, Kazuhiro Yamazaki, Hiroyoshi Ogasa

**Affiliations:** Department of Orthopedic Surgery, Hagi Civil Hospital 3460-3 Tubaki Hagi City Yamaguchi 758-0061 Japan

**Keywords:** Computer-assisted surgery, Femoral trochanteric fracture, Fluoroscopy based navigation, Operative time, Improving surgical efficiency

## Abstract

*Introduction*: In recent years, computer-assisted surgery has made it possible to undergo surgery with a high degree of precision. This study aimed to investigate the usefulness of computer-assisted surgery for femoral trochanteric fractures using the ADAPT (ADAptive Positioning Technology) system. *Methods*: A total of forty patients with femoral trochanteric fracture underwent intramedullary nailing for fracture fixation: in twenty patients, the ADAPT system (ADAPT group), and in the other twenty, it was not used (control group). The operative time, intraoperative fluoroscopy time, tip apex distance (TAD), and tip to head surface distance (TSD) were measured and compared between the two groups to assess the efficiency and accuracy of the surgery. *Results*: The operative time was significantly shorter (*P* < 0.05), intraoperative fluoroscopy time was significantly reduced (*P* < 0.01), and implant placement was significantly better in the ADAPT group (*P* < 0.01). *Conclusion*: Navigation systems have been developed to improve the efficiency of surgery. The ADAPT system was considered a very useful device for intramedullary nailing of femoral trochanteric fractures, as it reduced the intraoperative fluoroscopy time and improved the accuracy of implant placement, also reducing the operative time.

## Introduction

In recent years, navigation systems have increased, and their usefulness in orthopedic surgery is becoming increasingly important. Computer-assisted surgery (CAS) has been in development since the early 1990s. Thirty years on, CAS technology has been put to practical use in joint replacement, spinal surgery, tumor surgery, and trauma surgery, contributing to improved surgical accuracy [1]. However, orthopedic surgeons around the world are cautious about accepting CAS [2]. One of the main reasons for this is the increase in operative time [3]. There have been some reports of osteosynthesis of femoral trochanteric fractures using a navigation system, but although it helped to reduce radiation exposure and to insert the implant accurately, it was not efficient due to the time-consuming preoperative preparation and intraoperative manipulation [4, 5]. In particular, the ADAPT system (Stryker Leibinger GmbH & Co. KG, Freiburg, Germany) (Supplementary File 1) has been developed, which is expected to overcome this problem. ADAPT is a fluoroscopy-based navigation system that uses two-dimensions (2D) X-ray images taken with a C-arm device to construct three dimensions (3D) information. This computer-assisted surgery system has been described in detail in a previously published cadaver study [6]. In intramedullary nail surgery for femoral trochanteric fractures, the lag screw must be inserted in the ideal position [7]. ADAPT is capable of displaying intraoperative distances from the screw tip to the femoral head surface, such as the tip apex distance (TAD) and tip to head surface distance (TSD) proposed by Baumgaertner et al. [8]. ADAPT is not yet widely used, but its usefulness is gaining attention. There have been no reports of CAS for femoral trochanteric fractures with a reduction in operative time compared to conventional surgery. In this study, we evaluated the efficiency and accuracy of ADAPT and examined its usefulness.

## Materials and methods

A total of forty patients in the study agreed to undergo computed tomography (CT) before participating in the study. There were four male and thirty-six female patients, with a mean age of 85.7 years. Fracture classification was carried out using the Jensen classification [9]. There were six cases of Type 1, fifteen cases of Type 2, thirteen cases of Type 3, four cases of Type 4, and two cases of Type 5 (Table 1). Institutional Review Board (IRB) approval was obtained through the hospital for all patient surgeries in this study.

**Table 1:** Patient background. Percentages and ranges are shown for categorical and continuous data, respectively, in brackets.

	All (*n* = 40)	ADAPT group (*n* = 20)	Control group (*n* = 20)
Mean age (range)	85.7 (65–97)	85.9	83.5
Gender (%)			
Male	4 (10.0)	2 (10.0)	2 (10.0)
Female	36 (90.0)	18 (90.0)	18 (90.0)
Jensen classification (%)			
Type 1	6 (15.0)	2 (10.0)	4 (20.0)
Type 2	15 (37.5)	6 (30.0)	9 (45.0)
Type 3	13 (32.5)	9 (45.0)	4 (20.0)
Type 4	4 (10.0)	2 (10.0)	2 (10.0)
Type 5	2 (5.0)	1 (5.0)	1 (5.0)

### Surgical technique

**Figure 1:**
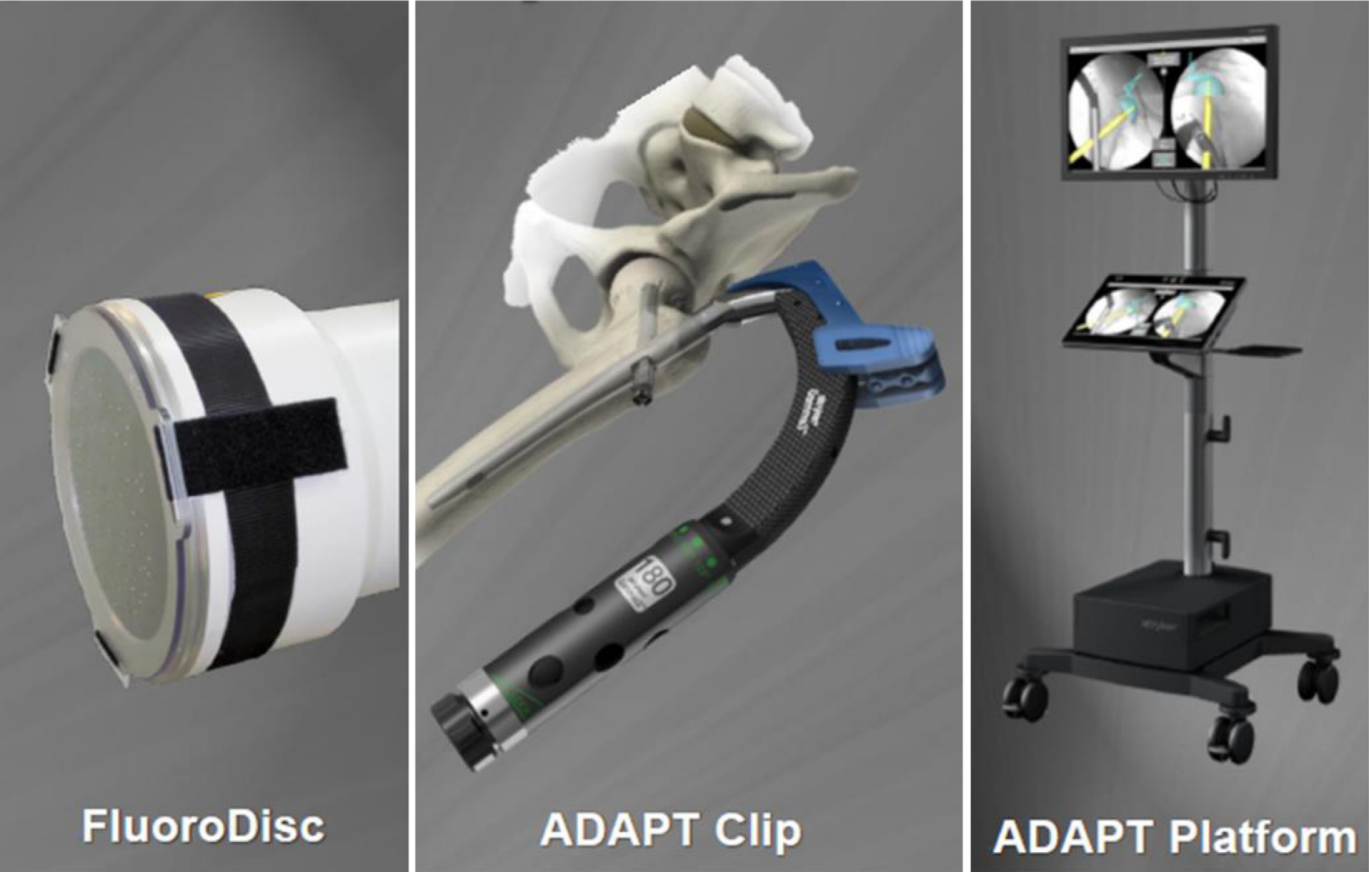
*FluoroDisc of ADAPT* – C-arm tube with FluoroDisc with position markers. *ADAPT Clip* – An ADAPT clip with an integrated position marker is attached to the Gamma3 targeting device. *ADAPT Platform* – ADAPT Platform detects and displays the position of the screw according to the positional markers.

All cases were operated on a fracture surgery traction table, and the implants were Gamma3 nails (Gamma3, Stryker, USA) (Supplementary File 2). When using ADAPT, it is necessary to attach the FluoroDisc to the C-arm device, the ADAPT clip to the Gamma3 targeting device, and use the ADAPT Platform as an additional monitor (Figure 1). When the C-arm is connected to the ADAPT Platform by cable, the position of the lag screw can be seen on the monitor in real-time during the operation. The ADAPT system is easy to fit and connect and does not require additional time, especially for preoperative preparation. The operation was undergone according to the usual technique for intramedullary nailing of femoral trochanteric fractures.

### ADAPT

**Figure 2:**
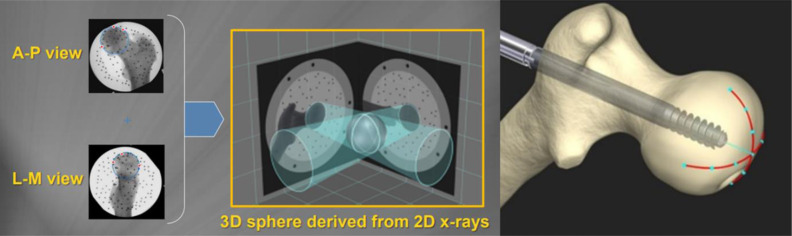
*Principle:* A 3D model is constructed by analyzing position markers obtained from 2D X-ray images in two directions. *Concept of TSD*: TSD is the distance from the tip of the screw to the surface of the femoral head.

**Figure 3:**
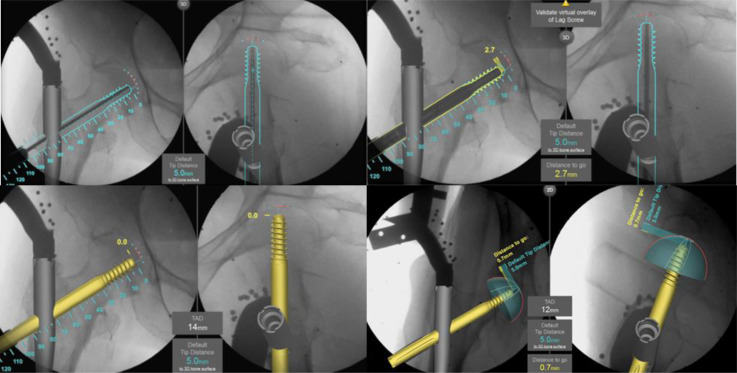
After inserting the guidewire along the outline displayed on the ADAPT monitor, the predicted position and length of the lag screw are displayed in a setting where the TSD is 5 mm. When the lag screw is actually inserted, the display shows how many more mm it needs to be inserted before the TSD will be in the 5 mm position. In addition, TAD can be checked at any time during the operation. After the lag screw has been installed, the position of the lag screw is displayed in 3D.

ADAPT calculates 3D information from the positional information obtained from the two-way fluoroscopic view of the C-arm device, automatically divides the femoral head, and detects the center of the head and the tip of the screw. The software creates a 3D model of the femoral head to determine the appropriate guidewire insertion direction and lag screw length for efficient implant placement, resulting in optimized TAD and TSD values. The TSD concept is designed to measure the 3D distance in the axial direction of the lag screw from the tip of the lag screw to the surface of the femoral head (Figure 2). First, after the two-way radiography with the C-arm device is completed, the monitor displays a 3D image of the nail, the center of the bone head, the surface of the bone head, and the direction of guidewire insertion. Next, when the guidewire is inserted, an outline of the lag screw, with the tip 5 mm from the surface of the femoral head, is displayed to confirm the position and length of the lag screw to be placed. After drilling, the lag screw is actually inserted, and the distance from the tip of the lag screw to the surface of the femoral head is indicated in 0.1 mm increments with each radiograph. Once the lag screw has been inserted, the position of the lag screw within the femoral head is displayed in 3D, and the TAD and TSD values are indicated on the monitor (Figure 3). The distal screw and end cap are then inserted as normal, and the operation is completed.

### Clinical assessment

Operative time, intraoperative fluoroscopy time, and accuracy of lag screw placement were assessed. We checked the position of the lag screws using plain radiographs and CT. The operative time, intraoperative fluoroscopy time, and TAD measured by postoperative X-p were compared between the two groups. TAD was measured using the method proposed by Baumgaertner et al. [8]. The actual 3D position of the lag screw within the femoral head was assessed by postoperative CT scan, and the TSD of the intraoperative data obtained by ADAPT was compared with the TSD measured postoperatively using CT. Two orthopedic surgeons and one radiologist measured the TAD and TSD twice, and the mean values were calculated.

### Statistical analysis

Analysis of the data was undertaken using XLSTAT add-in software on Microsoft Excel and SPSS statistical software. Values of *P* < 0.05 were considered to be statistically significant.

## Results

### Operative time

**Figure 4:**
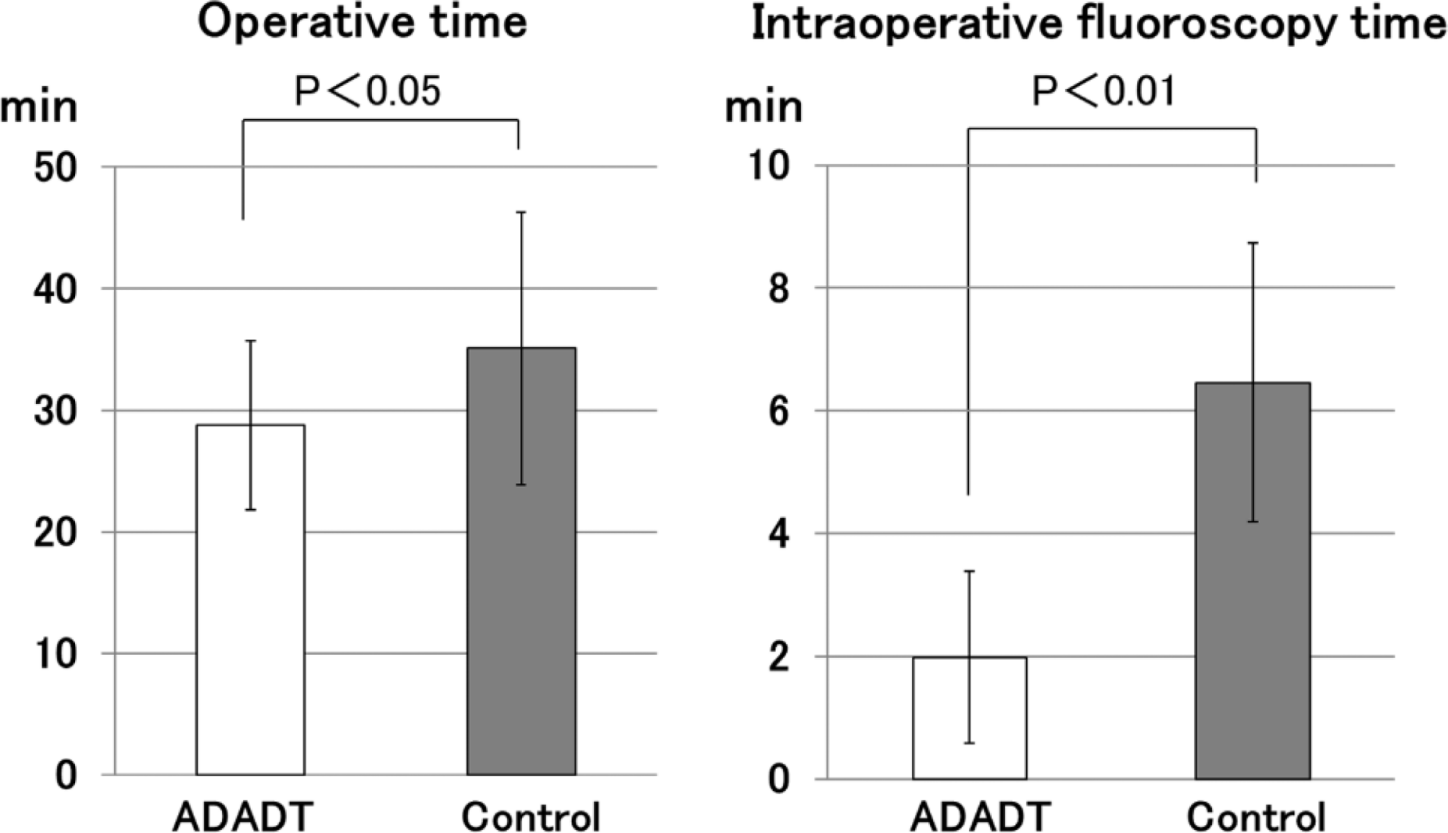
The operative time was significantly shorter in the ADAPT group. The intraoperative fluoroscopy time was significantly reduced in the ADAPT group.

The operative time was significantly shorter in the ADAPT group (*P* < 0.05). The operative time was 28.3 ± 6.99 min in the ADAPT group and 35.1 ± 11.2 min in the Control group (Figure 4).

### Intraoperative fluoroscopy time

The intraoperative fluoroscopy time was significantly reduced in the ADAPT group (*P* < 0.01). The intraoperative fluoroscopy time was 1.98 ± 1.40 min in the ADAPT group, and 6.46 ± 2.27 min in the Control group (Figure 4).

### Tip-apex distance (TAD)

**Figure 5:**
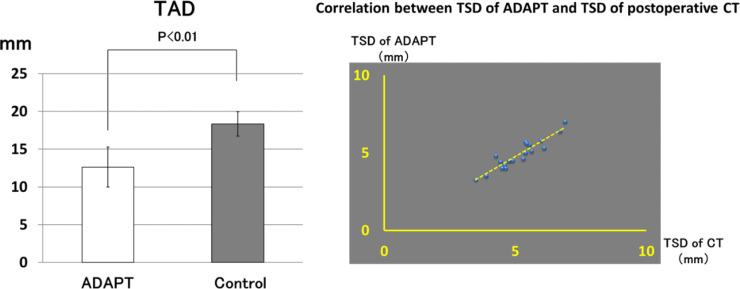
*TAD*: TAD significantly better in the ADAPT group. *Correlation between TSD of ADAPT and TSD of postoperative CT*: The correlation coefficient between the two groups is 0.81 (*r* = 0.81).

TAD was significantly better in the ADAPT group (*P* < 0.01). TAD was 12.6 ± 2.64 mm in the ADAPT group and 18.3 ± 1.63 mm in the Control group (Figure 5).

### Tip-to-head-surface distance (TSD)

The accuracy of the TSD displayed by ADAPT was high. TSD of ADAPT was 5.10 ± 0.87 mm, and the TSD of postoperative CT was 5.12 ± 0.88 mm. The mean absolute error of the TSD between the two groups was 0.49 mm (range, 0.25–0.79 mm). The correlation coefficient between the two groups is 0.81 (*r* = 0.81) (Figure 5).

## Discussion

Conventional navigation systems improve the accuracy of implant placement but are time-consuming and require a long time to set up [5, 10–12]. Atesok and Schemitsch conclude that the advantages of computer-assisted trauma surgery include increased accuracy and reduced radiation dose, but the disadvantages include increased operating time [13]. Our results show that the use of the ADAPT system improves the accuracy of implant placement and reduces radiation exposure and reduces the operative time. ADAPT has many intraoperative advantages. Using ADAPT, it is possible to plan the direction of guidewire insertion, the length of the screw and determine its position and entry point according to the outline displayed on the monitor. As a result, the number of times the guidewire is inserted and the time taken to measure the length of the screw can be reduced. In particular, the number of guidewire insertions for the lag screw was almost one time, making the operation more efficient and thus shortening the operation time. Before introducing ADAPT, we used to operate with two C-arms fixed in front and side positions. This method contributed to shortening the operation time, as it did not require the time to change the angle of the C-arm, compared to the operation with one C-arm, but it had the disadvantage of a small working space, which made the operation difficult, and it did not reduce the radiation exposure.

There are two limitations to this study. The first was that all the operations were undergone by experienced orthopedic surgeons. Secondly, there was always a trained computer operator present during the operation using ADAPT. Valsamis et al. state that the presence of a highly skilled surgeon and a trained computer operator in hip arthroplasty using a navigation system will certainly reduce the operative time [14]. This limitation may have contributed to the shortest learning curve and consequently to the shortest operative time.

The position of the lag screw has been reported to be important in the osteosynthesis of femoral trochanteric fractures [7]. One of the risk factors for cut-out complications after osteosynthesis is the position of the lag screw within the femoral head [15]. A widely known method for assessing cut-out risks is the TAD proposed by Baumgaertner et al. [8]. TAD is a useful criterion for cut-out risks, the ideal value being 5 mm < TAD < 20 mm [16, 17]. Herzog et al. concluded that the use of ADAPT resulted in a significant reduction in TAD and a more optimal positioning of the lag screw [18]. Our results also showed a significant reduction in TAD with the use of ADAPT.

TAD is measured from 2D images and TSD from 3D images. It was impossible to determine TAD intraoperatively, as TAD was originally a criterion to assess postoperative radiographs. It is difficult to determine TSD intraoperatively because TSD is a criterion that is assessed in 3D. However, ADAPT can demonstrate TAD and TSD intraoperatively. In particular, when inserting a lag screw, the TSD can be displayed on the screen in 0.1 mm increments for each X-ray exposure. The position of the lag screw within the bone head is converted from 2D to 3D by ADAPT, allowing the TSD to be determined intraoperatively. Takai et al. and Kuhl and Beimel compared the intraoperative TAD values of the ADAPT system with the TAD values on postoperative radiographs and reported the high accuracy of the intraoperative measurements [19, 20]. Our results comparing the intraoperative TSD values of the ADAPT system with the TSD values on postoperative CT also show the high accuracy of the intraoperative measurements. These results suggest that the ADAPT system is a computer-aided system with high accuracy of intraoperative measurements. The ADAPT system was considered to be a very useful device for intramedullary nailing of femoral trochanteric fractures, as it not only reduced the intraoperative fluoroscopy time and improved the accuracy of implant placement but also reduced the operative time.

## Supplementary Material

Supplementary material is available at https://www.sicot-j.org/10.1051/sicotj/2021056/olm *Supplementary File 1*. SICOT-J ADAPT.mpg *Supplementary File 2*. G3_IN_1.mpeg4.ac3.avi

## Conflict of interest

T.M. certifies that he has no financial conflict of interest in connection with this article. K.Y. certifies that he has no financial conflict of interest in connection with this article. H.O. certifies that he has no financial conflict of interest in connection with this article.

## Funding

This research did not receive any specific funding.

## Ethical approval

Ethical approval was not required.

## Informed consent

This article does not contain any studies involving human subjects.

## Authors contributions

T.M. was involved in the conception and coordination of the study and contributed to the acquisition, analysis and interpretation of the data and drafted the manuscript.

K.Y. and H.O. assisted in the conception and coordination of the study and helped with data analysis and preparation of the manuscript.
